# Predictive value of enhanced corneal biomechanical parameters for ectasia progression

**DOI:** 10.1007/s10384-024-01149-0

**Published:** 2025-01-20

**Authors:** Shizuka Koh, Renato Ambrósio, Ryota Inoue, Michael W. Belin, Naoyuki Maeda, Kohji Nishida

**Affiliations:** 1 Department of Innovative Visual Science, Osaka University Graduate School of Medicine, Room E7, 2-2 Yamadaoka, Suita, Osaka 565-0871 Japan; 2 Department of Ophthalmology, Osaka University Graduate School of Medicine, 2-2 Yamadaoka, Suita, Osaka 565-0871 Japan; 3 Instituto de Olhos Renato Ambrósio/Visare Personal Laser, Department of Ophthalmology, Federal University of the State of Rio de Janeiro (UNIRIO), Rio de Janeiro, Brazil; 4 SEED Co., Ltd, 2-11 Kanda Nishikicho, Chiyoda-ku, Tokyo, 101-0054 Japan; 5 Department of Ophthalmology and Vision Science, University of Arizona, Tucson, AZ USA; 6 Kozaki Eye Clinic, Osaka, Japan

**Keywords:** Keratoconus, Ectasia progression, Corneal biomechanics, Prediction

## Abstract

**Purpose:**

To determine whether corneal biomechanical parameters can predict ectasia progression.

**Study design:**

Retrospective observational study.

**Methods:**

The baseline corneal biomechanical parameters of 64 eyes of 41 young patients (age, < 25 years at the first visit) who were diagnosed with keratoconus (KC) or suspected KC at Osaka University Hospital and followed up for more than two years were reviewed. Suspected KC was defined as borderline cases with no definitive clinical or topographical abnormalities in both eyes. The eyes were divided into progressed (P) and non-progressed (NP) groups using the ABCD grading system of Scheimpflug-based tomography. The Scheimpflug-based corneal biomechanical parameters evaluated included deformation amplitude ratio within 2 mm, integrated radius, Ambrósio relational thickness to the horizontal profile, stiffness parameter at the first applanation, stress–strain index, E-staging, and Corvis Biomechanical Index. The optimized tomographic/biomechanical index (TBIv2), Belin/Ambrósio Enhanced Ectasia Deviation (BAD-D), and inferior-superior axial steepening values from Scheimpflug-based tomography were also evaluated.

**Results:**

Twenty-three and 41 eyes were categorized into the P and NP groups, respectively. Logistic regression analysis showed that age, BAD-D, and TBIv2 could predict ectasia progression. The specificity, sensitivity, and area under the receiver operating characteristic curve (AUROC) values for BAD-D combined with age were 0.82, 0.60, and 0.83, respectively, whereas those for TBIv2 combined with age were 0.65, 0.82, and 0.82, respectively.

**Conclusions:**

Baseline TBIv2 is a potentially useful predictive marker for ectasia progression in young patients, whereas baseline BAD-D could be used for establishing a definitive diagnosis.

**Supplementary Information:**

The online version contains supplementary material available at 10.1007/s10384-024-01149-0.

## Introduction

Corneal ectasia occurs and progresses due to complex interactions between environmental and mechanical forces, such as eye rubbing or atopy, and the inherent genetic susceptibility of the patient to ectasia [1–4]. The onset of keratoconus (KC) occurs during the early teenage years, and patients younger than 17 years old have a significantly elevated risk for KC progression [5]. The -manifestation and progression of KC are variable and often asymmetric between the two eyes. Diagnosis of KC is straightforward if patient shows noticeable clinical signs or abnormalities in Placido corneal topography maps. However, if a patient does not show any abnormal clinical and topographical signs but KC or a predisposition to KC is suspected due to refractive error values, increased astigmatism, or family history, accurate diagnosis may be challenging, even after objective evaluation using multiple methods including corneal tomography. The absence of internationally standardized diagnostic criteria and severity classifications for KC makes it difficult to assign appropriate diagnostic names and severity levels in clinical practice.

Recently, corneal biomechanical assessments have been used for early and efficient detection of KC and corneal ectasia. Using combined biomechanical parameters, such as the tomographic biomechanical index (TBI), a combined parameter based on Scheimpflug tomographic and biomechanical assessments, is reported to be a sensitive method for detecting even the mildest forms of ectasia [6]. Moreover, a recent study indicated that the optimized tomographic biomechanical index (TBIv2) enhances the detection of ectasia [7]. However, little is known about the predictive value of enhanced biomechanical parameters for ectasia progression. Therefore, the aim of this study was to investigate whether corneal biomechanical measurements can predict ectasia progression in a young population.

## Patients and methods

### Study design and ethics statements

This was a retrospective observational study of young patients with KC or suspected KC who were younger than 25 years at the initial visit and were followed up for more than two years. This study was conducted in accordance with the tenets of the Declaration of Helsinki. This study was approved by the Institutional Review Board/Ethics Committee of Osaka University Hospital (registration number: 09297-20). All the patients provided informed consent after receiving an explanation of the nature and possible outcomes of the study.

## Characterization of ectasia progression

The enrolled patients were selected from patients who underwent ophthalmological examinations at Osaka University Hospital between October 2021 and September 2022 and were diagnosed with KC or suspected KC. We included patients who were younger than 25 years at the initial visit and had completed at least two years of uneventful follow-up. KC was defined as the presence of signs of KC in at least one eye on slit lamp examination or topographic signs of KC at the first visit. Suspected KC was defined as absence of clinical and topographic KC signs bilaterally at the first clinical visit, referred by a primary eyecare provider to a university hospital for more comprehensive examination. Reasons for referral to university hospitals included severe astigmatism, progressive astigmatism, left-right asymmetry in refractive power, and a family history of KC. Observation of Fleischer ring, Vogt striae, corneal thinning, and/or corneal protrusion on slit lamp examination was considered indicative of KC. Topographic KC signs on the anterior corneal surface, such as abnormal localized steepening or an asymmetric skewed bow-tie pattern [2] were assessed using Placido disk corneal topography (TMS-5; Tomey Corporation). The typical topography for suspected KC was confirmed based on the following: a KC screening performed using Placido disk corneal topography, a 0% Klyce/Maeda Keratoconus Index and 0% Smolek/Klyce Keratoconus Severity Index [8, 9], and an inferior-superior asymmetry value (IS value) < 1.4 at 6 mm [10] on the topographic map. Posterior corneal changes were not included. The exclusion criterion was history of ocular surgery or other ocular pathologies, including corneal scarring or acute hydrops. One or both eyes of each patient were included depending on the case.

Scheimpflug-based corneal tomographic (Pentacam HR; Oculus Optikgeräte GmbH) and biomechanical (Corvis ST; Oculus Optikgeräte GmbH) assessments of the cornea were performed at baseline and at each follow-up visit by experienced examiners. All patients were examined at least twice to obtain well-focused and properly aligned ocular images. The enrolled eyes were classified into progressed (P) or non-progressed (NP) groups using on the ABCD grading system of the Pentacam HR [11], which assesses the anterior radius of curvature (A), posterior radius of curvature (B), corneal pachymetry at the thinnest position (C), and distance best corrected vision (D). Each parameter is independently staged from 0 to 4 (Table 1). The classification into the P or NP group was conducted by one of the authors, who was blinded to the results of the biomechanical assessments. In all cases, the differential map between the first visit and last visit was reviewed and used for classification. The criterion used for classification of patients into the P group was a significant change or unquestionable progression from one of the four (A, B, C, or D) grades. That is, if the stage progresses by one level (becomes more severe) or if there is evidence of progression on the differential map, even within the same stage, it was included in the P group.

**Table 1:** ABCD grading system [11]

ABCD criteria	A: ARC (3 mm zone)	B: PRC (3 mm zone)	C: Thinnest pach (µm)	D: BDVA
Stage 0	7.25 mm (< 46.5 D)	> 5.90 mm	> 490 μm	≥ 20/20 (≥ 1.0)
Stage 1	7.05 mm (< 48.0 D)	> 5.70 mm	> 450 μm	< 20/20 (< 1.0)
Stage 2	> 6.35 mm (< 53.0 D)	> 5.15 mm	> 400 μm	< 20/40 (< 0.5)
Stage 3	> 6.15 mm (< 55.0 D)	> 4.95 mm	> 300 μm	< 20/100 (< 0.2)
Stage 4	6.15 mm (> 55.0 D)	< 4.95 mm	≤ 300 μm	< 20/400 (< 0.05)

*ARC* anterior radius of curvature, *PRC* posterior radius of curvature, Thinnest pach, corneal pachymetry at the thinnest position, *BDVA* distance best corrected vision

## Assessment of the predictive value of corneal biomechanical measurements for ectasia progression

The following biomechanical parameters obtained from the Corvis ST were selected for the analysis: deformation amplitude ratio within 2 mm (DAR2mm), integrated radius (IR), Ambrósio relational thickness to the horizontal profile (ARTh), stiffness parameter at the first applanation (SPA1), stress–strain index (SSI), E-staging, and Corvis Biomechanical Index (CBI). The definitions of these parameters are as follows: DAR2mm, the ratio between the deformation amplitude measured at the apex and 2 mm; IR, the area under the inverse concave radius curve between the first and second applanations; ARTh, thickness profile in the temporal–nasal direction; SPA1, stiffness parameter at the first applanation; E-staging, KC staging based on biomechanical response; and SSI, the entire stress–strain curve of the cornea. The CBI incorporates the dynamic corneal response parameters obtained from the device. In addition to these parameters, the Belin/Ambrósio Enhanced Ectasia Deviation (BAD-D) and IS values from the Pentacam HR and the TBIv2 from the Corvis ST were evaluated.

### Statistical analysis

Comparisons of age and tomographic and biomechanical parameters at baseline between non-progressed and progressed groups were performed by the Wilcoxon rank-sum test. The first correlation analysis of the above-mentioned parameters included age at baseline. A scatterplot matrix was used to identify the factors that influenced the prediction of ectasia progression. Considering their clinical relevance, these factors were incorporated into the model. The variance inflation factors were calculated to check for multicollinearity. Logistic regression analysis was performed to determine the predictive factors for ectasia progression. To investigate the diagnostic value of the factors correlated with ectasia progression, the area under the receiver operating characteristic curve (AUROC) of each factor was calculated, and the factors with the highest accuracy, sensitivity, and specificity were determined. Statistical significance was set at *p* < 0.05. The R software (version 4.1.0; R Foundation for Statistical Computing, Vienna, Austria) was used for the correlation analysis. Other analyses were conducted using MedCalc for Windows, version 22.110 (MedCalc Software).

## Results

We enrolled 64 eyes of 41 patients (22 men, 19 women) with KC or suspected KC who were younger than 25 years old at the first clinical visit and were followed up for a minimum of two years. The age of the patients at initial examination was 18.9 ± 3.2 years. All the patients were Japanese. Data obtained from questionnaires on the presence of known KC-related risk factors in the 41 patients are summarized in Table 2.

**Table 2:** Presence of Keratoconus risk factors (based on data obtained from questionnaires)

Risk factors	KC (*n* = 26 %)	Suspected KC (*n* = 15 %)
Family history of KC	1 (3.8)	5 (33.3)
Atopy	8 (30.8)	3 (20.0)
Asthma	5(19.2)	1 (6.7)
Allergy	14 (53.8)	6 (40.0)
Eye rubbing	20 (76.9)	6 (40.0)
Prone sleeping position	6 (23.1)	2 (13.3)

*KC* keratoconus

### Categorization of ectasia progression

**Fig. 1:**
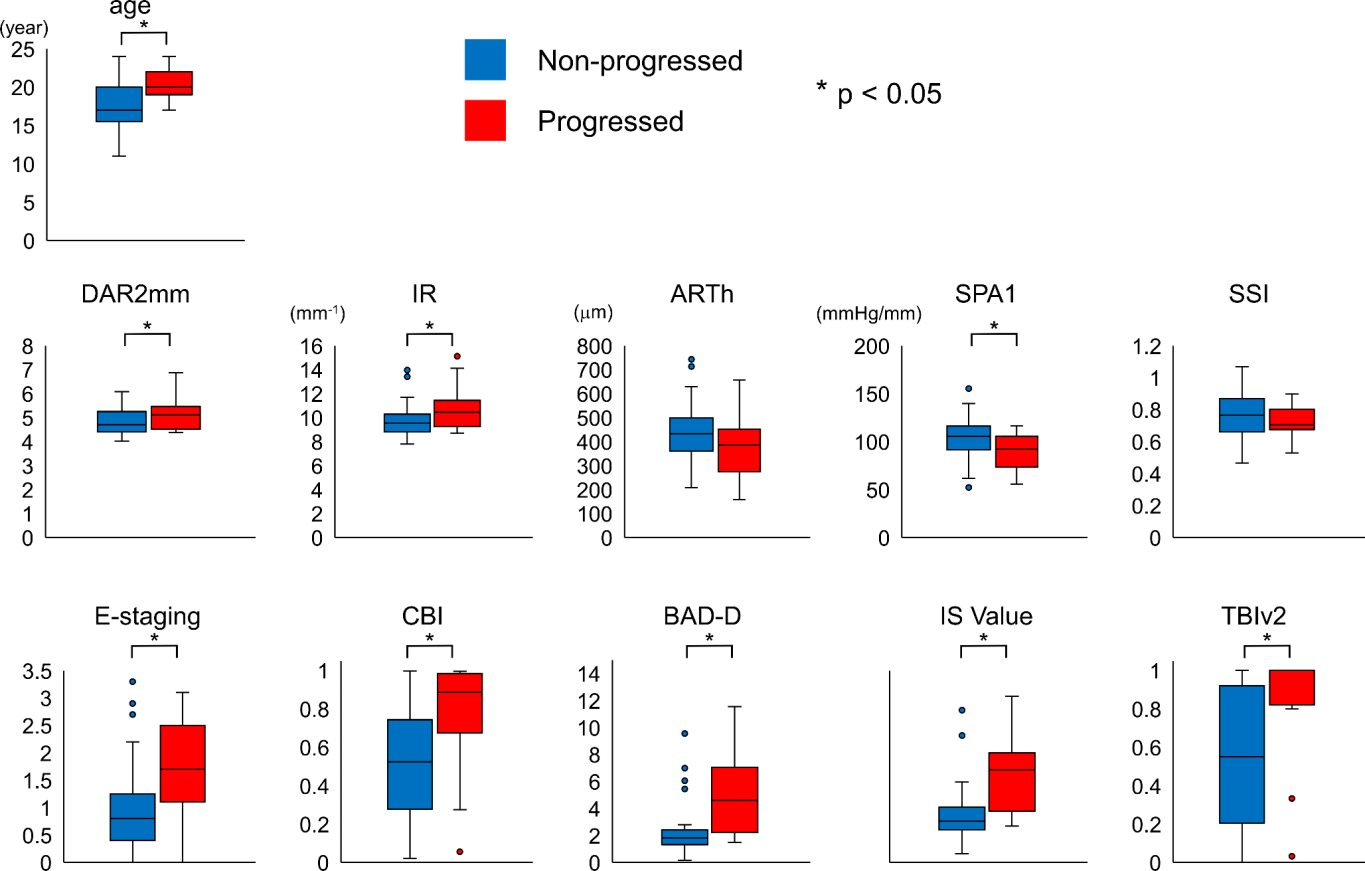
The distributions of the baseline tomographic and biomechanical parameters for both groups (Non-progressed and Progressed)

In two patients, one eye was classified as P and the other as NP. Both eyes of the remaining patients were classified into the same group. Overall, 23 eyes of 17 patients were classified as P, whereas 41 eyes of 26 patients were classified as NP. The baseline tomographic and biomechanical features of eyes in both groups (P and NP) are shown in Fig. 1 and summarized in the **Online Resource**. All the enrolled patients were diagnosed with KC (at least one eye with KC) or suspected KC at baseline. However, the enrolled eyes were classified into three groups based on the baseline status of each eye: (1) the eye of the bilateral ectasia which had at least KC signs at slit lamp or Placido topography bilaterally, (2) the fellow eye with normal topography in very asymmetric ectasia having clinical KC or at least topographic KC signs in one eye, and (3) the eye from the patients who had no clinical and topographic KC signs bilaterally. Table 3 shows the outcomes (P or NP) of the three groups categorized according to the status of each eye. In the group with bilateral ectasia, progression was observed in 58% of patients based on the tomography grading system. Progression was also observed in the eyes without clinical or topographic signs of KC; however, the percentage was small compared with that of eyes with bilateral ectasis.

**Table 3:** Outcomes based on the status of each eye (progressed or non-progressed)

Group	Number of eyes	Progressed %	Non-progressed %
Bilateral ectasia	29	17 (58.6)	12 (41.4)
Fellow eye with normal topography in a case of very asymmetric ectasia	5	1 (20.0)	4 (80.0)
Eyes with borderline KC without bilateral clinical and topographic KC signs	30	5 (16.7)	25 (83.3)

*KC* keratoconus

## Predictive value of corneal biomechanical parameters for ectasia progression

**Fig. 2:**
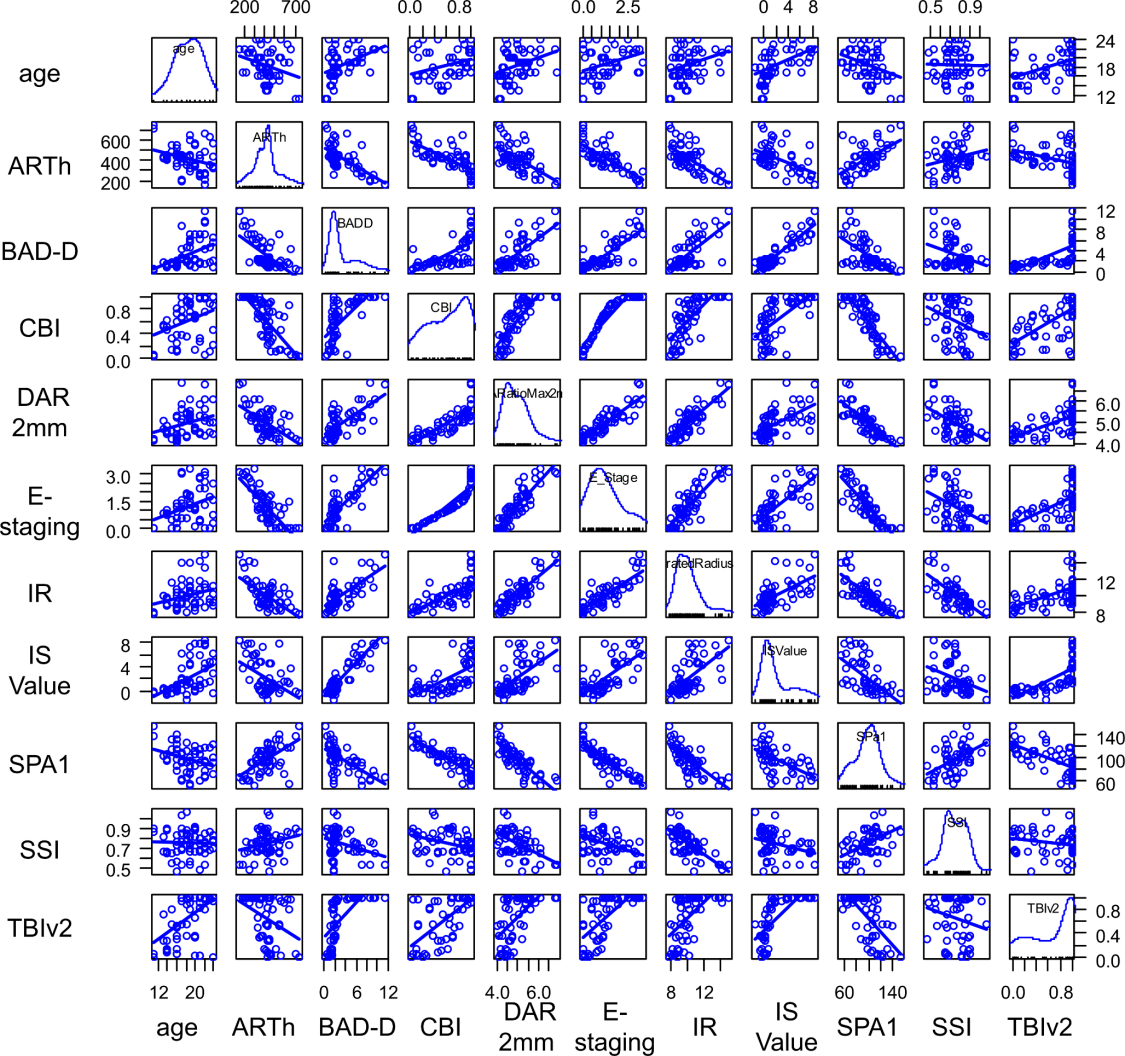
Scatterplot matrices of the 11 baseline tomographic and biomechanical parameters analyzed in this study

**Fig. 3:**
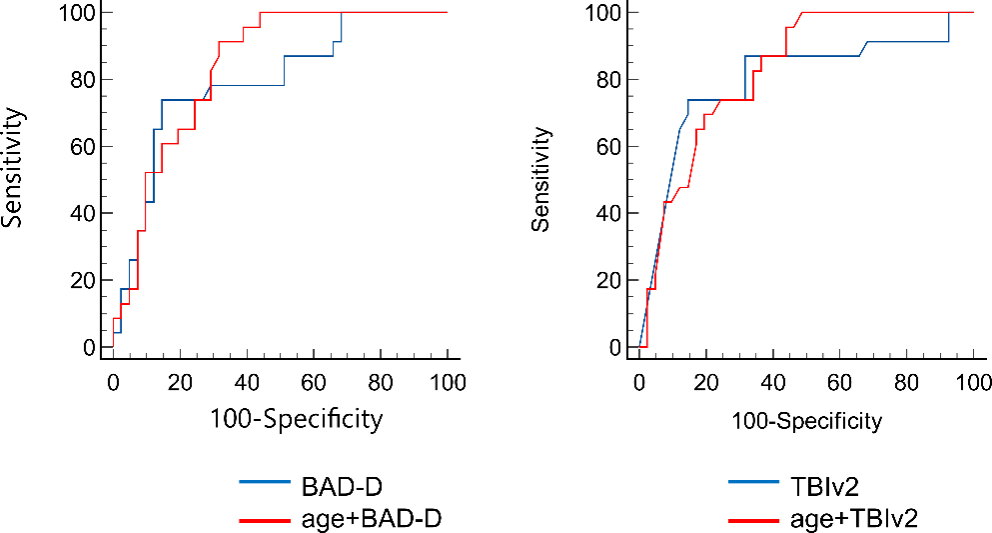
Receiver operating characteristic curves indicating the predictive ability of ectasia progression

Figure 2 shows the scatterplot matrices of the 11 analyzed baseline parameters. Age, BAD-D, CBI, SSI, and TBIv2 were associated with ectasia progression. Logistic regression analysis showed that age, BAD-D, and TBIv2 were predictive factors for ectasia progression. (Table 4) Regarding the predictive ability of each factor, BAD-D combined with age had the highest AUROC value (0.830), with 60% sensitivity and 82% specificity, whereas the AUROC for BAD-D alone was 0.794. The AUROC for TBIv2 combined with age was 0.823, with 82% sensitivity and 65% specificity, whereas the AUROC for TBIv2 alone was 0.797 (Fig. 3).

**Table 4:** Results of logistic regression analysis

Variable	*p*	Odds ratio	95% CI
age	0.021	1.687	1.082 to 2.630
BAD-D	0.035	18.990	1.226 to 294.064
age	0.041	2.410	1.038 to 5.594
TBIv2	0.043	10.1 × 10^8^	1.898 to 532.494 × 10^15^
age	0.035	2.005	1.051 to 3.825
CBI	0.077	24.3 × 10^6^	0.161 to 3.673 × 10^15^
age	0.166	2.735	0.658 to 11.371
SSI	0.354	10.9 × 10^6^	0.000 to 8.515 × 10^21^

*BAD-D* Belin/Ambrósio enhanced ectasia deviation, *TBIv2* optimized tomographic/biomechanical index *CBI* Corvis Biomechanical Index, *SSI* stress–strain index

## Discussion

This retrospective observational case series demonstrated that that age, BAD-D, and TBIv2 could predict ectasia progression in young patients with KC or suspected KC. According to a recent meta-analysis [5], young patients have a significantly increased risk for KC progression. Therefore, early detection and frequent follow-up for prompt initiation of appropriate interventions are necessary for these patients.

The biomechanical properties of the cornea have been studied, particularly for the detection of subclinical KC and ectatic corneal disease. However, little is known about long-term corneal biomechanical changes in unoperated eyes with KC. In our recent study that involved the evaluation of corneal biomechanical changes over three years, we found that in patients with very asymmetric ectasia, corneal softening may occur in the fellow eye having normal topography and not normal tomography [12]. Moreover, TBIv2 has been recently reported to enhance ectasia detection [7]. Based on these findings, we hypothesized that evaluating corneal biomechanical properties could facilitate the prediction of ectasia progression. Separating the KC group (including “forme fruste keratoconus” (FFKC)) from the “suspected KC” group (patients at risk but currently unaffected) would help draw clearer conclusions. However, in studying progressive diseases such as KC, with a high risk of progression in young individuals, focusing initially on young patients across all groups, as we did in this study, is clinically meaningful to determine “whether progression occurs.” Therefore, we included all groups in the present study. The results of the present study indicate that in young individuals, baseline TBIv2 may be a potentially useful prognostic marker of ectasia progression, whereas baseline BAD-D can be used for establishing a definitive diagnosis.

Previously, assessment of the progression of KC was based on clinical parameters [13–15]. Advanced corneal imaging techniques have improved screening, diagnosis, classification, and severity staging for clinical follow-up in cases of KC and ectatic corneal diseases [16, 17]. The progression of KC is now more commonly documented using corneal topography or corneal tomography [18–23]. However, little is known about the prognostic evaluation of the risk of progression. In the present study, BAD-D combined with age had high specificity (82%), whereas TBIv2 combined with age had high sensitivity (82%). BAD-D is useful for establishing a definitive diagnosis because of its high specificity in patients confirmed to have KC. Moreover, it may be useful in decision-making regarding the next treatment steps for patients confirmed to have KC. On the other hand, TBIv2 is highly sensitive, and although it is associated with a higher number of false positives than BAD-D, it is effective and can be used to confirm a negative test result. From this perspective, TBIv2 may be useful for predicting the progression of KC. Earlier awareness of the risk of ‘silent’ progression of KC would be helpful for detecting subtle progressive changes during clinical monitoring. A recent study with a one-year follow-up period, which was conducted using molecular biology testing, indicated that the level of interleukin-13 in combination with nerve growth factor in tears can predict the progression of KC [24]. The pathogenesis of KC is related to a combination of genetic, biomechanical, biochemical, and environmental risk factors, including inflammation [1–3, 25]. As multimodal imaging tools are fundamental for corneal assessments in patients with KC, multimodal prognostic evaluations, such as molecular biology testing and tomographic and biomechanical assessment, could be useful for predicting the progression of KC in the future. However, future studies with a large population of younger participants of different ethnicities are warranted to verify the predictive potential of these assessment methods for KC.

We enrolled eyes with KC of varying severities, ranging from very mild to advanced KC, and borderline cases of suspected KC in this study. We found that 58% of eyes with bilateral KC showed progression. This finding is comparable to that of a study conducted in Scotland [26], which indicated that 41% of young patients with KC (mean age 18, range 14–21) showed progression over a period of four years. In patients with very asymmetric ectasia and clinical signs of KC in one eye, the fellow eye with normal topography is referred to as FFKC. Although there is no unified definition of FFKC, the most widely used definition in various studies is “fellow eye of a clinical KC eye that has no clinical or topographic signs of KC” [27]. However, if the other ectatic eye has topographic KC signs without clinical signs, it cannot be termed ‘FFKC’. Notably, precise terminologies for describing these conditions are lacking. Considering that having “typical corneal topography” is included as a criterion in the revised Amsler–Krumeich classification [28], we specifically defined in the present study “the fellow eye with normal topography in a case of very asymmetric ectasia with the other eye showing clinical KC signs or at least topographic KC signs.” An improved classification system that considers the current diagnostic tools should be established.

In the present study, the patients with suspected KC showed no clinical or topographic KC signs bilaterally. The details of the optical characteristics of borderline KC are unknown. It is possible that some cases are close to “bilateral FFKC” [29]. Recently, we reported that patients with borderline suspected KC had significantly greater total higher-order aberrations and coma from the posterior corneal surface and whole eye than normal controls [30]. However, the heterogeneity of the study population is one of the limitations of the study. Some of the patients had severe astigmatism, whereas those who visited the hospital for a thorough examination because of a family history of KC did not necessarily have a high degree of astigmatism. The frequency of eye rubbing (76.9%) was higher in the young patients enrolled in this study than that (58.5%) from KC across all age group in our previous study [31]. Nevertheless, careful follow-up is recommended for these patients even if KC was initially excluded. In addition, a more extensive case series that includes a sub-analysis performed according to the type and amount of astigmatism would be interesting.

This study has some limitations. A retrospective study of young patients with KC or suspected KC who were followed for more than two years might be biased. Patients who subsequently had no complaints or were deemed non-progressive may not have been followed. The ABCD grading system alone would not be used to determine progression. However, it is useful to investigate the variation in clinical features of KC. Therefore, we used ABCD grading system in this study. Future studies using another classification systems should be considered. Some patients used soft or rigid gas-permeable contact lenses. However, owing to the retrospective nature of the study, the duration of contact lens discontinuation before examinations varied among patients. Ideally, patients should have discontinued contact lens use for a prolonged, standardized period before testing. However, it is often challenging for patients with KC to do so, as discontinuing lens wear could significantly impair their quality of life and vision. The current study was a single-center study with a small sample size. A follow-up multicenter study with a larger sample size would be ideal for characterizing the progression of ectasia in the Japanese population. Even though the patients in the suspected KC group were clinically suspected to have KC, the possibility of “eyes with corneal shape abnormalities that are not KC” could not be ruled out. Further longitudinal follow-up and multimodal assessments are essential for improving the predictive ability of the studied parameters for ectasia progression.

In conclusion, this study demonstrated that enhanced corneal biomechanical parameters may predict ectasia progression. Clinical follow-up of young patients with KC or suspected KC for more than two years showed that baseline BAD-D and TBIv2 have similar predictive values for ectasia progression but can be used differently. Baseline TBIv2 could aid the detection of ectasia susceptibility and identify patients with a risk for ectasia progression, mainly in cases of suspected but excluded KC if there are no abnormal topographical and clinical signs. Baseline objective tomographic analysis, such as that for deriving BAD-D, should still be considered for confirming the diagnosis.

## Electronic supplementary material

Below is the link to the electronic supplementary material.

Supplementary Material 1
