# Supplementary material for: Predictive value of enhanced corneal biomechanical parameters for ectasia progression
Source: Jpn J Ophthalmol. 2025 Jan 20;69(2):174–81. doi: 10.1007/s10384-024-01149-0 (PMC12003495; doi:10.1007/s10384-024-01149-0)
Supplement: Supplementary file 1 — Supplementary Material 1 [file 10384_2024_1149_MOESM1_ESM.pdf]

Online Resource (Supplementary table)

Baseline tomographic and biomechanical features of eyes in both groups

Group	Non-progressed (NP)	Progressed (P)	p
Age	20.00±2.00<br>20.00 (17 to 24)	17.66±3.38<br>17.00 (11 to 24)	<0.01*
DAR2mm	5.21±0.67<br>5.12 (4.38 to 6.88)	4.82±0.54<br>4.70 (4.02 to 6.08)	0.03*
IR (mm^{-1})	10.74±1.58<br>10.45 (8.69 to 15.11)	9.81±1.45<br>9.54 (7.80 to 14.23)	<0.01*
ARTh (μm)	380.38±122.03<br>385.69 (157.62 to 656.74)	439.77±112.29<br>432.80 (208.45 to 743.65)	0.08
SPA1 (mmHg/mm)	88.51±18.36<br>92.08 (55.75 to 116.42)	103.61±22.01<br>105.63 (52.30 to 155.38)	<0.01*
SSI	0.73±0.10<br>0.71 (0.53 to 0.90)	0.76±0.14<br>0.77 (0.47 to 1.07)	0.49
E-staging	1.65±0.90<br>1.70 (0.00 to 3.10)	0.97±0.80<br>0.80(0.00 to 3.30)	<0.01*
CBI	0.75±0.28<br>0.89 (0.06 to 1.00)	0.53±0.29<br>0.53 (0.02 to 1.00)	<0.01*
BAD-D	5.00±2.80<br>4.61 (1.49 to 11.56)	2.31±1.91<br>1.81 (0.17 to 9.55)	<0.01*
IS Value	1.25±2.18<br>0.57 (-1.46 to 7.65)	3.60±2.57<br>3.80 (0.28 to 8.39)	<0.001*
TBIv2	0.86±0.29<br>1.00 (0.03 to 1.00)	0.54±0.35<br>0.55 (0.00 to 1.00)	<0.001*

Data are shown as the mean±SD.

*Statistically significant ($p < 0.05$).

ARTh, Ambrósio relational thickness to the horizontal profile; BAD-D, Belin/Ambrósio Enhanced Ectasia Deviation; CBI, Corvis Biomechanical Index; DAR2mm, deformation

amplitude ratio within 2 mm; IR, integrated radius; IS value, inferior–superior asymmetry value, SPA1, stiffness parameter at first applanation; SSI, stress–strain index; TBIv2, tomographic/biomechanical index.
